# Enzymatically Triggered Peptide–Lipid Conjugation of Designed Membrane Active Peptides for Controlled Liposomal Release

**DOI:** 10.1021/acsomega.4c01387

**Published:** 2024-04-18

**Authors:** Alexandra Iversen, Johanna Utterström, Robert Selegård, Daniel Aili

**Affiliations:** Laboratory of Molecular Materials, Division of Biophysics and Bioengineering, Linköping University, Linköping 581 83, Sweden

## Abstract

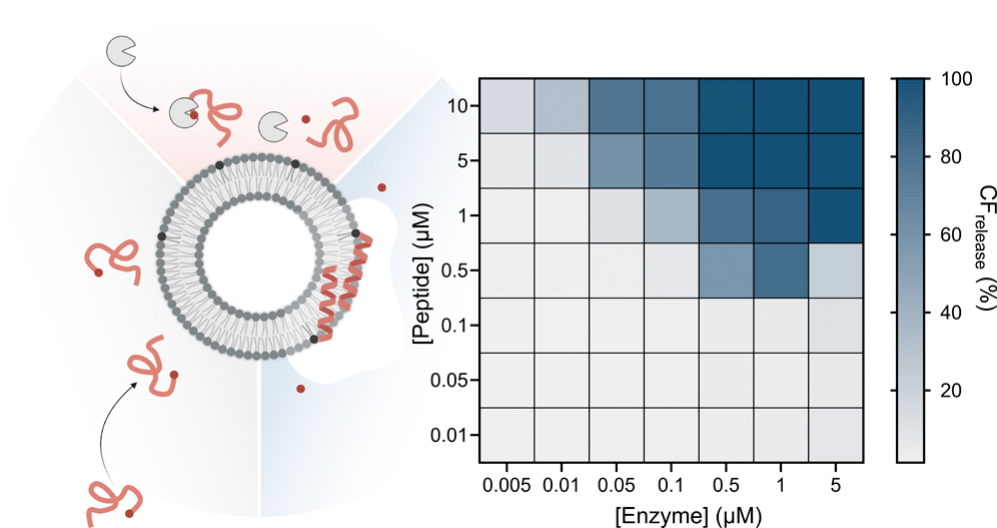

Possibilities for controlling the release of pharmaceuticals from liposomal drug delivery systems can enhance their efficacy and reduce their side effects. Membrane-active peptides (MAPs) can be tailored to promote liposomal release when conjugated to lipid head groups using thiol-maleimide chemistry. However, the rapid oxidation of thiols hampers the optimization of such conjugation-dependent release strategies. Here, we demonstrate a *de novo* designed MAP modified with an enzyme-labile Cys-protection group (phenylacetamidomethyl (Phacm)) that prevents oxidation and facilitates *in situ* peptide lipidation. Before deprotection, the peptide lacks a defined secondary structure and does not interact with maleimide-functionalized vesicles. After deprotection of Cys using penicillin G acylase (PGA), the peptide adopts an α-helical conformation and triggers rapid release of vesicle content. Both the peptide and PGA concentrations significantly influence the conjugation process and, consequently, the release kinetics. At a PGA concentration of 5 μM the conjugation and release kinetics closely mirror those of fully reduced, unprotected peptides. We anticipate that these findings will enable further refinement of MAP conjugation and release processes, facilitating the development of sophisticated bioresponsive MAP-based liposomal drug delivery systems.

## Introduction

Liposomal drug delivery systems can improve efficacy and safety of therapeutics.^1^ Depending on their composition, drug delivery systems can promote localized delivery of the active pharmaceutical ingredient to target tissues, resulting in lower risk of severe side effects.^1^ Liposomal drug delivery systems often display excellent biocompatibility and long circulation times.^2^ They are also biodegradable^3^ and can carry both hydrophilic and lipophilic therapeutic substances.^4^ About 14 liposomal drug formulations have been approved and are available on the market for treatment of *e.g.*, breast cancer, ovarian cancer, meningitis, fungal infections, and leukemia.^5^ Albeit improving the toxicity profile of the drugs,^6^ their bioavailability in the target tissue can suffer from low release rates from the liposomes. Strategies for controlling the release profiles using liposomes responsive to light, heat, magnetic fields, or pH have been widely explored but have not yet rendered any clinical success.^7^ Possibilities to use membrane active peptides (MAPs) to trigger release of liposomal content have also been explored with promising results.^8−10^

MAPs are a broad group of peptides that includes both antimicrobial peptides (AMPs) and cell-penetrating peptides (CPPs), which interact with lipid membranes causing perturbations in lipid membrane integrity.^11^ MAPs display a large chemical, structural, and functional diversity, but are often relatively short (<40 amino acids) with an abundance of cationic amino acids (Arg and Lys) and they typically lack defined secondary structure unless associated with a lipid membrane.^12^ Many MAPs fold into an amphipathic α-helix, where the hydrophobic face of the helix enables interactions with the hydrophobic core of the lipid bilayer^12^ supported by electrostatic interactions between cationic residues and polar and negatively charged lipid head groups.^13^ The interaction of MAPs with lipid bilayers, including cell membranes, can result in disruption of lipid membrane integrity due to pore formation, lipid membrane thinning, or lipid dissolution. The peptide–lipid-bilayer interactions are also highly dependent on lipid composition, peptide concentration, pH, and temperature.^13^ AMPs and CPPs are rarely selective and can cause hemolytic and cytotoxic effects even at moderate concentration, and thus are typically not ideal triggers for liposomal release. Wimley and coworkers managed to improve selectivity and performance of a natural antimicrobial peptide (melittin) for liposomes comprised of 1-palmitoyl-2-oleoyl-sn-glycero-3-phosphocholine (POPC), a common lipid in drug formulations, by generating and screening large rational combinatorial libraries for potent pore-forming peptides.^14^ Mizukami et al. developed another strategy for localized and triggered liposomal release by modifying the antimicrobial peptide temporin with a branch that comprised a substrate sequence of caspase-3, which drastically reduced the membrane activity of the peptide.^15^ Cleavage of the branching sequence by caspase-3 resulted in a regain of function. Srivastava and coworkers have developed a collagen mimetic lipopeptide that can be inserted in liposomal membranes and trigger content release when cleaved by matrix metalloproteinase 9 (MMP-9).^10^ However, since the rate and extent of release of the liposomal content depend on the mismatch between the acyl chains of the synthesized lipopeptide and phospholipid components of the liposomes, the release rate was slow and required high concentrations of lipopeptides (30 mol%) and unphysiological protease concentrations.

We have previously demonstrated several lysine-rich amphipathic *de novo* designed MAPs that lack membrane activity on lipid vesicles unless covalently coupled to maleimide headgroup-functionalized lipids *via* the thiol-moiety in a single Cys residue.^8,9,16,17^ The *in situ* lipidation promotes peptide accumulation on the vesicles, resulting in peptide folding and lipid membrane partitioning, triggering the efficient release of encapsulated compounds. The membrane activity can be tuned by varying the number of heptad repeats of the peptides^8^ and the lipid composition of the vesicles.^9^ The membrane activity can also be tuned by introducing complementary peptides, designed to heterodimerize with the MAP and fold into coiled coils or helix–loop–helix four helix bundles.^8,16^ Dimerization competes with membrane partitioning, which effectively prevented premature release of the liposomal content even after conjugation of the MAPs to the liposomes. Proteolytic cleavage of the complementary peptide resulted in the recovery of membrane activity of the MAP and thus release of the encapsulated compounds.^17^ The multiple possibilities available for tuning the release kinetics are attractive and can facilitate development of advanced bioresponsive drug delivery systems. However, the strategy suffers from difficulties in controlling the conjugation of the peptides to the vesicles due to the rapid oxidation of the Cys residues during the preparation of a drug delivery system. The Michael addition reaction between maleimides and thiols is a well-studied coupling strategy that has been used in a wide range of applications,^18,19^ including synthesis of the antibody-drug conjugates brentuximab vedotin and trastuzumab emtansine.^20,21^ However, thiols oxidize under ambient conditions, which effectively prevents their reaction with maleimides.^22^ The *in situ* lipidation process thus becomes less efficient and may result in nonoptimal surface concentrations of the MAP.

**Scheme 1:**
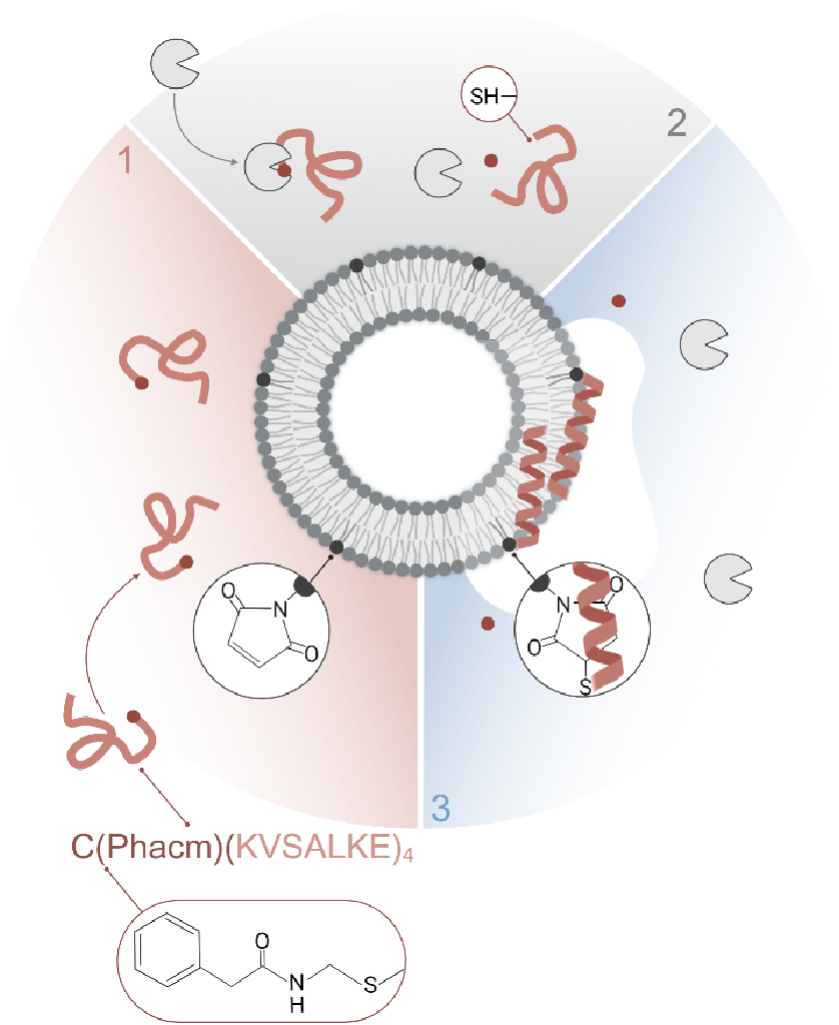
Schematic Illustration of Enzyme Triggered Peptide–Lipid Conjugation to Maleimide-Functionalized 95:5 POPC:MPB Liposomes: 1) C(Phacm)KV_4_ Peptides Carrying the Thiol-Protection Group Phacm on the N-Terminal Cysteine Are Inactive and Unfolded Prior Deprotection, 2) Addition of PGA Results in Cysteine Deprotection Exposing a Free Thiol, and 3) Deprotected C(Phacm)KV_4_ Reacts with the Maleimides Resulting in Peptide Folding and Lipid Membrane Partitioning and Liposome Cargo Release

Here, we show the possibility of preventing oxidation of the thiol moiety using an enzyme-labile thiol protection group that drastically facilitates the peptide–lipid conjugation process under ambient conditions (Scheme 1). The *de novo* designed MAP CKV_4_ was synthesized with a N-terminal Cys residue protected by phenylacetamidomethyl (Phacm), rendering the peptide C(Phacm)KV_4_. The Phacm-protection group is compatible with both Fmoc and Boc solid-phase peptide synthesis and can be selectively deprotected using the enzyme penicillin G acylase (PGA) under physiological conditions.^23^ The deprotection process is cytocompatible^24^ and rapid,^25^ leaving free thiols^23^ accessible for conjugation to thiol-reactive lipids. The release of carboxyfluorescein (CF) from the vesicles was used as an indicator of successful MAP conjugation. Addition of PGA to C(Phacm)KV_4_ in the presence of maleimide-functionalized vesicles consequently resulted in a rapid and folding dependent CF release. The release rate could be tuned by both the peptide and PGA concentration. No release was seen in the absence of PGA or by PGA alone. The possibility to prevent the oxidation and tailor the conjugation process of CKV_4_ to vesicles can further facilitate the development of more elaborate MAP-controlled drug delivery systems.

## Results and Discussion

### Enzymatically Mediated Peptide–Lipid Conjugation

**Figure 1:**
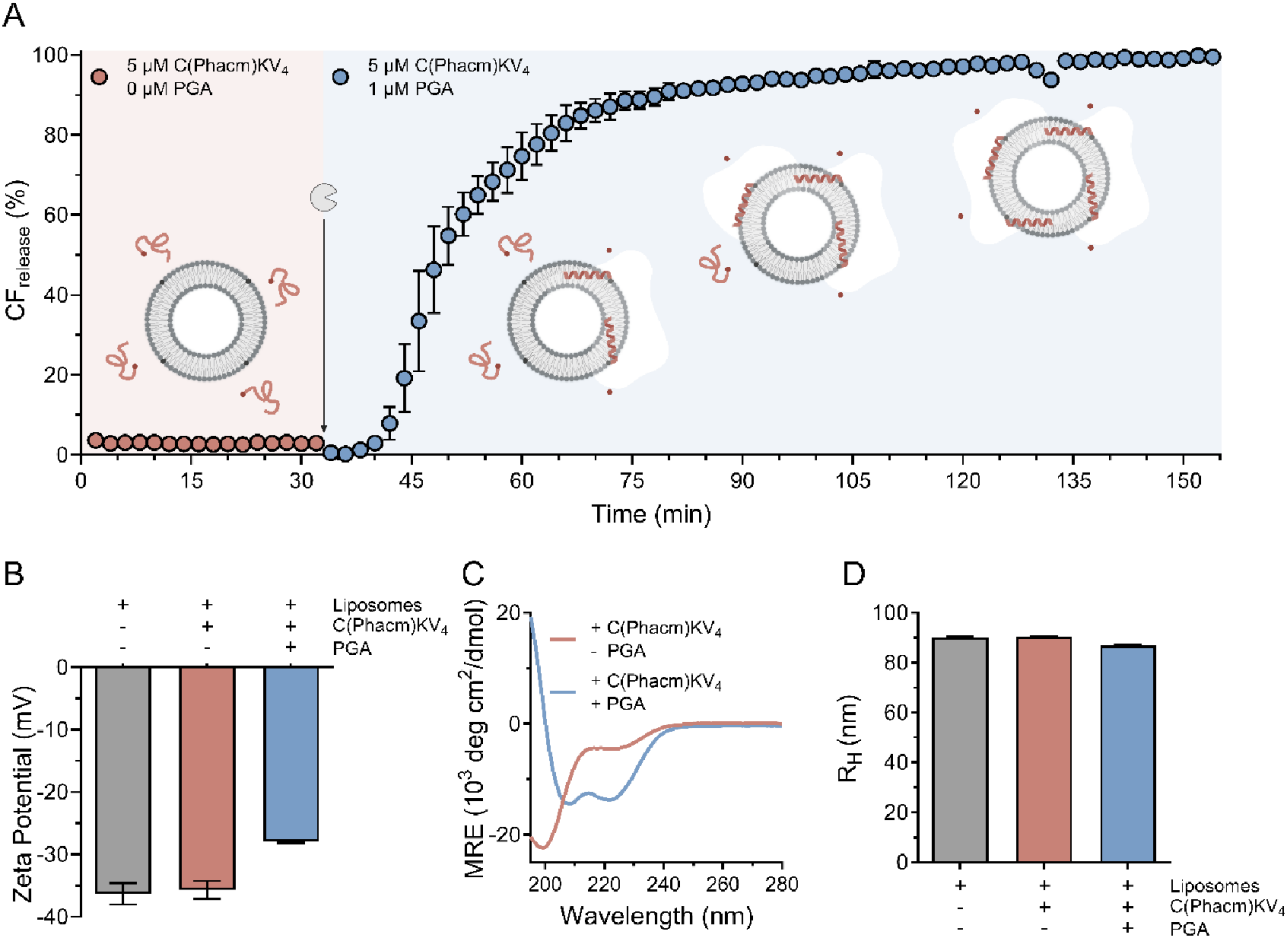
A) CF release kinetics of 5 μM C(Phacm)KV_4_ incubated with 40 μM 95:5 POPC:MPB vesicles (pink). After 30 min, 1 μM PGA was added (blue), and the release was followed for another 120 min. B) Zeta potential of 95:5 POPC/MPB vesicles: alone (gray), with 5 μM C(Phacm)KV_4_ (pink), and after 2 h incubation with 5 μM C(Phacm)KV_4_ and 0.5 μM PGA (blue). C) CD spectra of 30 μM C(Phacm)KV_4_ in 0.01 M PB with 1.2 mM 95:5 POPC:MPB vesicles, after 17.5 h of incubation with (blue) or without (pink) 0.15 μM PGA. The maleimide:peptide ratio was 2:1. D) Hydrodynamic radius (*R*_H_) of 95:5 POPC:MPB vesicles: alone (gray) or after 2 h incubation with 5 μM C(Phacm)KV_4_ (pink), and after 2 h incubation with 5 μM C(Phacm)KV_4_ and 0.5 μM PGA (blue). Error bars are the relative peak widths of the size distribution.

The cysteine (Cys) terminated peptide CKV_4_ (C-(KVSALKE)_4_) was designed to fold into a well-defined α-helix when covalently conjugated to maleimide headgroup-functionalized lipids *via* the Cys thiol group, as a result of lipid membrane partition folding coupling.^8^ However, oxidation of the thiol moiety in Cys impedes conjugation, which complicates both the handling of the peptides and the optimization of this strategy for the development of bioresponsive drug delivery systems. To prevent thiol oxidation, we modified Cys in CKV_4_ with an enzyme-labile protection group (Phacm), rendering the peptide C(Phacm)KV_4_ (Scheme 1). Cys(Phacm) is stable under a wide range of conditions and can be deprotected by penicillin G acylase (PGA) in aqueous solutions at physiological pH and ionic strength.^23^ To investigate the possibilities of removing the Phacm group using PGA to trigger lipid membrane conjugation of C(Phacm)KV_4_, we utilized carboxyfluorescine (CF) loaded liposomes. In the absence of PGA, no CF release was seen when incubating C(Phacm)KV_4_ with large unilamellar vesicles (LUVs) consisting of 5 mol% 1,2-dioleoyl-sn-glycero-3-phosphoethanolamine-N-[4-(p-maleimidophenyl)butyramide] (MPB-PE) and 95 mol% 1-palmitoyl-2-oleoyl-sn-glycero-3-phosphocholine (POPC) (95:5 POPC:MPB-PE) confirming that the thiol group was not reactive (Figure 1A). However, addition of 1 μM PGA triggered rapid CF release, indicating efficient Cys deprotection and subsequent lipid membrane conjugation of the peptide. Conjugation was further confirmed by the increase in the ζ-potential of the POPC/MPB-PE vesicles from −36 mV in the absence of PGA and C(Phacm)KV_4_ to −28 mV after addition of C(Phacm)KV_4_ and PGA due to high positive net charge (+4) of CKV_4_ at pH 7.4 (Figure 1B). No changes in ζ-potential were observed after the addition of C(Phacm)KV_4_ in the absence of PGA.

We have previously seen that the peptide-mediated membrane destabilization process is highly folding dependent. Circular dichroism (CD) spectra showed no defined secondary structure of C(Phacm)KV_4_, neither in the absence nor in the presence of LUVs (95:5 POPC:MPB-PE) prior addition of PGA, which clearly indicates that the protection group was stable under physiological conditions and prevented peptide–lipid interactions (Figures 1C, S1A). Addition of penicillin G acylase (PGA) resulted in a drastic change in the far UV CD spectra with characteristic minima at around 208 and 222 nm corresponding to an α-helical conformation (Figure 1C). The contributions of PGA secondary structure elements to the CD spectra were negligible (Figure S1B). The deprotection of the Phacm-group by PGA thus resulted in the folding of C(Phacm)KV_4_, which strongly indicates that the peptide was successfully conjugated to MPB-PE. The ratio of the mean residue ellipticity at 222 and 208 nm (MRE_222_/MRE_208_) increased from 0.5 prior to PGA addition to 1.0 after Phacm deprotection (Table 1), which indicates that most of the peptides existed as α-helices after lipid conjugation. The Phacm deprotection strategy did consequently not interfere with peptide-folding and lipid membrane partitioning.

**Table 1:** MRE Values at 208 and 222 nm and the Ratio of MRE_222_:MRE_208_ for C(Phacm)KV_4_ and C(Phacm)KV_4_ Incubated with PGA (CKV_4_) in the Presence of 95:5 POPC:MPB-PE Vesicles

Peptide	MRE_222_	MRE_208_	MRE_222_/MRE_208_
C(Phacm)KV_4_	–4.6	–10.5	0.5
CKV_4_	–14.3	–14.7	1.0

### Concentration Dependence and Release Kinetics of PGA Induced Peptide Triggered Release

**Figure 2:**
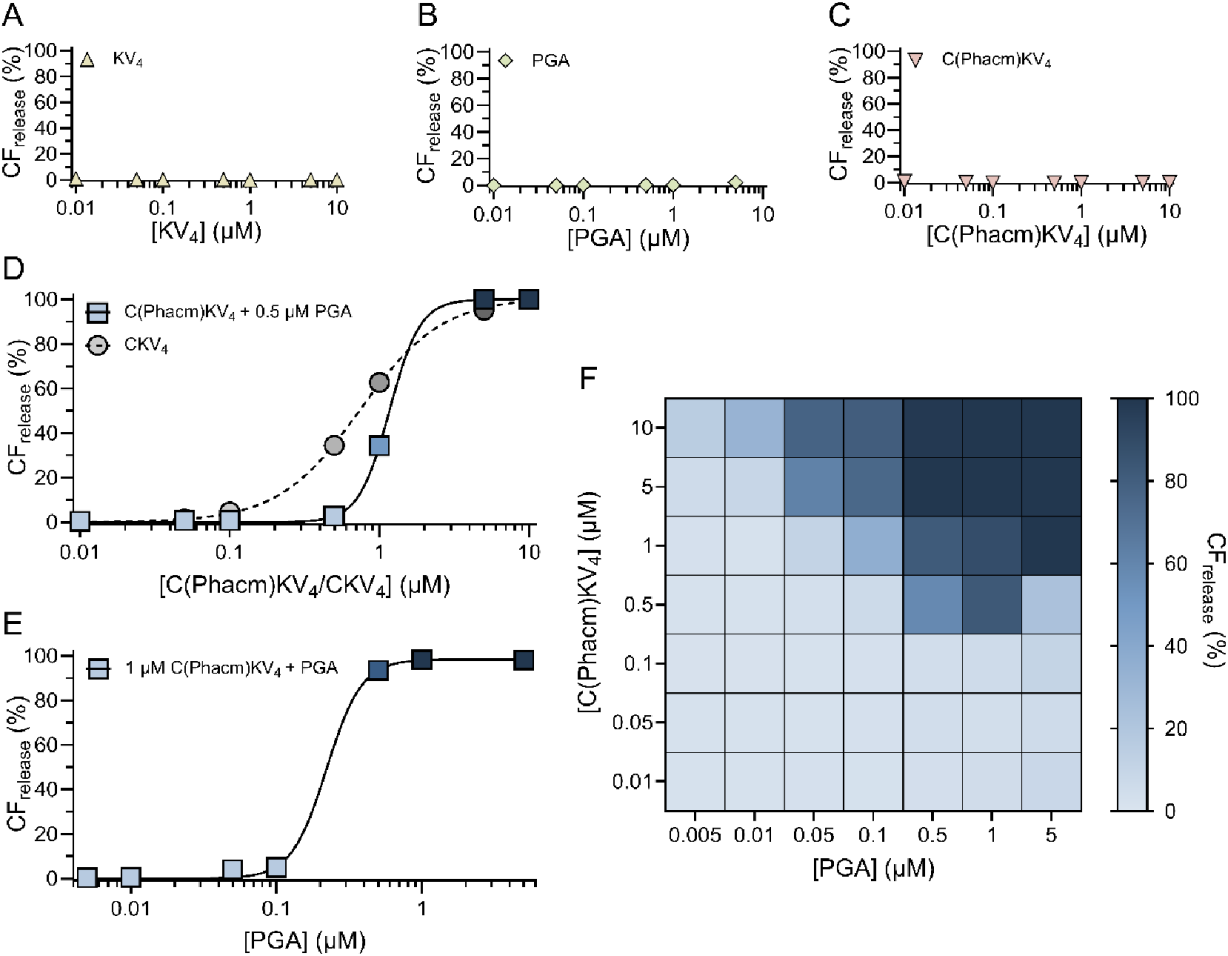
Total CF release from 40 μM 95:5 POPC:MPB vesicles after 2 h incubation with A) 0.01–10 μM KV_4_ (yellow triangles), B) 0.005–5 μM PGA (green diamonds), C) 0.01–10 μM C(Phacm)KV_4_ (pink triangles), D) 0.01–10 μM C(Phacm)KV_4_ and a fixed PGA concentration of 0.5 μM PGA (blue squares) and with 0.01–10 μM CKV_4_ (gray circles) respectively, and E) 0.005–5 μM PGA and a fixed C(Phacm)KV_4_ concentration of 1 μM (blue squares). In A–E) data were fitted to a Hill equation, *n* = 3. F) Heat map of the total CF release (%) achieved after 2 h with varying PGA and C(Phacm)KV_4_ concentrations. The colors represent the degree of release. *n* = 2.

No CF-release was obtained from 95:5 POPC/MPB-PE vesicles in the absence of peptide or PGA (Figure S2A) or when exposed to KV_4_ (*i.e.*, CKV_4_ lacking the Cys) (Figures 2A, S2B). Nor was any release caused by PGA alone (Figures 2B, S2C) or C(Phacm)KV_4_ prior to the addition of PGA (Figures 2C, S2D). However, when incubating C(Phacm)KV_4_ at varying concentrations with a fixed PGA concentration of 0.5 μM a clear C(Phacm)KV_4_ concentration dependent CF release was seen, similar to that of unprotected CKV_4_ (Figures 2D, S2E). The resemblance between the release profiles of CKV_4_ and PGA deprotected C(Phacm)KV_4_ indicates that at this concentration, PGA can fully and rapidly remove the Phacm group.

**Figure 3:**
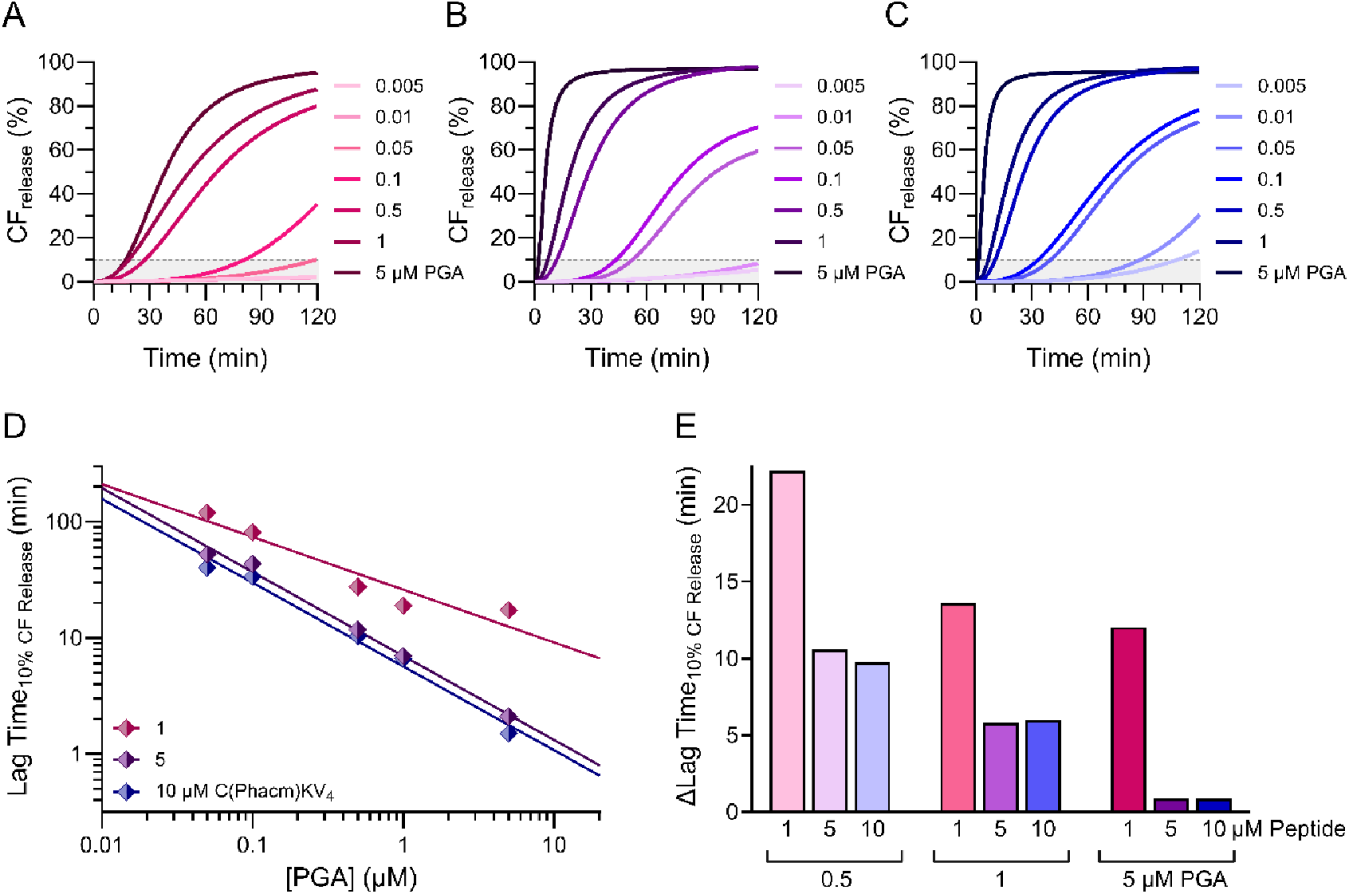
CF release kinetics from 40 μM 95:5 POPC:MPB vesicles during 2 h incubation with varying concentrations of PGA combined with A) 1 B) 5 and C) 10 μM C(Phacm)KV_4_. The dashed line and gray area underneath highlights release kinetics up to 10% CF release. Data were fitted to a Hill equation. D) The time required to reach 10% CF release from 40 μM 95:5 POPC:MPB vesicles when incubated with 1, 5, or 10 μM C(Phacm)KV_4_ combined with a range of PGA concentrations. Data were fitted to linear functions. E) The difference in lag time (ΔLag Time_10% CF release_) for 1, 5, and 10 μM unprotected CKV_4_ and C(Phacm)KV_4_ combined with 0.5, 1, or 5 μM PGA (Lag Time(CKV_4_) – Lag Time(C(Phacm)KV_4_ + PGA)).

After establishing that PGA could deprotect C(Phacm)KV_4_, we investigated the impact of PGA concentration on C(Phacm)KV_4_ triggered CF-release. A fixed concentration of C(Phacm)KV_4_ of 1 μM was first used to probe the full dynamic range of the enzyme (Figures 2E, S2F). Extensive CF release was seen for PGA concentrations above 0.1 μM after 2 h of incubation. At lower concentrations, the release rate was clearly limited by the lower number of deprotected C(Phacm)KV_4_. To gain a better understanding of how the two sequential reactions of deprotection and peptide–lipid conjugation were influencing the CF release process, the influence of varying both PGA and C(Phacm)KV_4_ concentration was explored (Figure 2F). Both increasing concentrations of C(Phacm)KV_4_ and PGA resulted in a more extensive release, confirming that it is the total amount of deprotected CKV_4_ that determines the final CF-release. Limited release was obtained for the three lowest C(Phacm)KV_4_ concentrations tested (0.01–0.1 μM) irrespective of the concentration of PGA, which is in line with previous observations that a critical concentration of peptides is required to efficiently disrupt lipid membrane integrity.^8^ However, the release process is highly time dependent. At lower PGA concentrations, the deprotection of C(Phacm)KV_4_ was clearly the rate limiting step. Limited release was seen until the concentration of deprotected peptides had increased above a threshold value, resulting in a distinct lag-phase prior to the subsequent CF burst release (Figures 3A-C, S3, S4). The lag time, defined here as the time from PGA addition until 10% CF release was observed, was dependent on the PGA concentration (Figure 3D). The higher the PGA concentration, the shorter the lag time. A similar lag-burst behavior has previously been described for destabilization of liposomes by phospholipases, where the gradual accumulation of lysolipids in the lipid membranes triggers a CF burst release above a certain threshold concentration.^26−28^ Here, the lag time reflects the time required for PGA to generate CKV_4_ above the threshold concentration required for triggering lipid membrane destabilization. At higher PGA concentrations, the deprotection was fast enough to reduce the lag phase, and the CF release profile became more similar to that of unprotected CKV_4_ (Figures 3E, S5).

The deprotection of the peptide-thiol moiety using PGA clearly simplifies the conjugation process of CKV_4_ to vesicles and eliminates the issues with thiol oxidation. In addition, this strategy provides a new means to modulate the release rate of encapsulated compounds and to explore the mechanisms involved in membrane destabilization by CKV_4_ and other similar selective membrane active peptides, facilitating the development of MAP-functionalized liposomal drug delivery systems.

## Conclusions

We have investigated the possibility to prevent thiol oxidation of the membrane active peptide CKV_4_ using the enzyme-labile Cys-protection group Phacm, which can be selectively deprotected by PGA. The protected peptide, C(Phacm)KV_4_, lacked a defined secondary structure, even in the presence of maleimide functionalized lipid vesicles, and was not able to trigger any release from vesicles encapsulating self-quenching concentrations of CF. However, upon deprotection of the Cys residue by PGA in the presence of lipid vesicles, CF release and peptide folding were observed demonstrating *in situ* lipidation and subsequent peptide–lipid membrane partitioning. Peptide conjugation did not cause any vesicle aggregation or micellization. Furthermore, the release could be tuned by both the PGA and C(Phacm)KV_4_ concentrations, but the rate-limiting step was clearly the amount of deprotected C(Phacm)KV_4_. At low concentrations of PGA (≤1 μM), a distinct lag-phase prior to CF release was thus observed. The lag-phase was eliminated by increasing the concentration above 1 μM PGA resulting in a release profile that was almost identical to fully reduced nonprotected CKV_4_. These results show that Phacm-protection of the Cys residue is an excellent method for preventing thiol oxidation of membrane active peptides that require thiol-dependent peptide–lipid conjugation. The possibilities to protect the Cys residue from oxidation and control the lipid conjugation process can facilitate the development of sophisticated, bioresponsive MAP-based drug delivery systems.

## Methods

### General

The amino acid Cys(Phacm) was purchased from Iris Biotech (Marktredwitz, Germany), and the lipids were purchased from Avanti Polar Lipids (Alabaster, Alabama). All other reagents were purchased from Merck (Merck, Darmstadt, Germany), PGA for instance, or Fischer Scientific (Hampton, New Hampshire, USA).

### Peptide Synthesis

Solid-phase peptide synthesis on a Liberty Blue Automated Microwave Peptide Synthesizer (CEM, Matthews, North Carolina) was used to synthesize the peptides KV_4_ (KVSALKEKVSALKEKVSALKEKVSALKE) and C(Phacm)KV_4_ (C(Phacm)KVSALKEKVSALKEKVSALKEKVSALKE). Rink Amide ProTide resin (LL) (CEM, Matthews, North Carolina) was used as a solid support to obtain peptides with a C-terminal amide. All couplings were performed twice under microwave conditions with a 5-fold amino acid excess, Oxyma pure as a base, and N,*N*′-diisopropylcarbodiimide (DIC) as a coupling reagent. The cysteine amino acid used for the synthesis of C(Phacm)KV4 was Fmoc-L-Cys(Phacm)–OH (Iris Biotech, Marktredwitz, Germany). After synthesis, the N-terminal of KV_4_ and C(Phacm)KV_4_ was acetylated. The crude peptides were cleaved from the solid support by treatment with trifluoroacetic acid (TFA)/H_2_O/triisopropylsilane (TIPS). Cold diethyl ether was used to concentrate and precipitate the crude peptides. After synthesis, the crude peptides were purified by RP-HPLC (Dionex/Thermo Fisher, Waltham, Massachusetts) with an aquatic gradient of acetonitrile (ACN) and 0.1% TFA. The purity was confirmed by analytical RP-HPLC with an aquatic gradient of ACN and 0.1% TFA (Figure S6A). Pure peptide sequences were verified by MALDI-ToF mass spectroscopy (Figure S6B). The peptide CKV_4_ (CKVSALKEKVSALKEKVSALKEKVSALKE) was synthesized with an acetylated N-terminal amide and a C-terminal amide by GL Biochem (Shanghai, China).

### Liposome Preparation

Small unilamellar liposomes were prepared by thin-film hydration and extrusion. POPC and MPB-PE in chloroform (Avanti Polar Lipids, Alabaster, Alabama) were mixed to achieve a ratio of 95:5 mol%. A dried lipid film was obtained by evaporating chloroform using a nitrogen stream and placing the film in a vacuum desiccator overnight to completely remove the solvent. The lipid film was hydrated with 0.01 M PB at pH 7.4 filtrated through a 0.2 μm filter or 50 mM carboxyfluorescein dissolved in 10 mM PB with 90 mM NaCl at pH 7.4. The lipid suspension was then placed on a shaking table for 10 min and vortexed for 1 min, resulting in a lipid concentration of 5 mg/mL. Monodisperse vesicles were obtained by extruding the suspensions 21 times through a 100 nm polycarbonate membrane using a mini extruder (Avanti Polar Lipids, Alabaster, Alabama). Prior to further analysis, unencapsulated CF was removed from lipid suspensions hydrated in CF-stock by size exclusion chromatography on a G-25 column (Cytiva, Marlborough, Massachusetts) in PBS.

### Carboxyfluorescein (CF) Release Assay

Liposome membrane integrity was studied by monitoring the fluorescence emitted from CF upon release from liposomes. CF was encapsulated in liposomes at self-quenching concentrations (50 mM) and fluorescence was observed over time using a 96-well plate in a fluorescent plate reader (Tecan Infinite M1000 Pro, Tecan Austria GmbH, Grödig/Salzburg, Austria) at room temperature with λ_ex_ = 485 nm and λ_em_ = 520 nm. Liposomes in PBS (10 mM, pH 7.4) were prepared, and the fluorescence was measured (*F*_0_). A peptide or enzyme was then added to the wells so that desired concentrations were obtained. PBS (10 mM, pH 7.4) volumes equal to that was added to the control wells. Control wells contained liposomes in PBS (10 mM, pH 7.4) and sample wells contained liposomes, peptide, and/or PGA (Merck, Darmstadt, Germany) in PBS (10 mM, pH 7.4). All of the wells had the same lipid concentration (40 μM). Fluorescence measurements were performed every other minute over the desired time span (*F*). After measurements, Triton X-100 was added to the wells to achieve a 1% Triton concentration. After 10 min of incubation to achieve complete liposome lysis, fluorescence was measured (*F*_tot_). Percentage of released CF was calculated according to CF release (%) = (*F* − *F*_0_)/(*F*_tot_ − *F*_0_) × 100.

### Circular Dichroism (CD)

CD measurements were recorded on a Chirascan (Applied Photophysics, Leatherhead, United Kingdom) at room temperature using a 1 mm path length quartz cuvette, and scanning was performed between 195 and 280 nm with steps of 0.5 nm. All samples were prepared in PB (10 mM, pH 7.4). Liposomes are nonchiral entities and will therefore not affect the data received from the measurements. Liposomes in PB were used as background for measurements of samples containing liposomes and peptide, whereas liposomes and enzyme in PB were used as background for measurements of samples containing liposomes, peptide, and enzyme. The lipid concentration used was 1.2 mM, the peptide concentration used was 30 μM (corresponding to a 1:2 peptide:maleimide ratio), and the enzyme concentration used was 0.15 μM. Measurements on samples with liposomes, peptide, and enzyme were performed after 17.5 h of incubation time. At least three spectra of each sample were recorded, averaged, and then smoothed using the Savitzky–Golay algorithm.

### Dynamic Light Scattering (DLS)

Hydrodynamic radius was determined by DLS at room temperature on an ALV/DLS/SLS-5022F system (ALV-GMBH, Langen, Germany) equipped with a 632.8 nm HeNe laser. Samples were prepared in PB (10 mM, pH 7.4) that was filtered through a 0.2 μm filter. Lipid concentration was 50 μM, peptide concentration was 5 μM, and enzyme concentration was 0.5 μM. The correlation curves of 10 consecutive 30 s runs were averaged and used to obtain the distribution of particle size.

### Zeta Potential

Measurements of ζ-potential were performed using a Malvern ZetaSizer Nano ZS90 instrument (Malvern Panalytical, Malvern, Worcestershire, United Kingdom). Samples were prepared in PB (10 mM, pH 7.4) that was filtered through a 0.2 μm filter. Lipid concentration was 650 μM, peptide concentration was 65 μM, and enzyme concentration was 6.5 μM.

### Data Fitting

Data from CF release assays were fitted to a Hill slope according to *Y* = (*B*_max_ × *X*^h^)/(*K*_d_^h^ + *X*_h_). Lag phase data were fitted to a linear curve according to *Y* = *Y*_Intercept_ + Slope × *X*. CD data were smoothened using the Savitzky–Golay algorithm.

## Supporting Information Available

Additional CD spectra of C(Phacm)KV_4_ and PGA, CF release kinetics data, analytical HPLC chromatogram of C(Phacm)KV_4_ and KV_4_, and MALDI-TOF MS data of C(Phacm)KV_4_ and KV_4_ (PDF)
