# Supplementary material for: Enzymatically Triggered Peptide–Lipid Conjugation of Designed Membrane Active Peptides for Controlled Liposomal Release
Source: ACS Omega. 2024 Apr 18;9(17):19613–9. doi: 10.1021/acsomega.4c01387 (PMC11064179; doi:10.1021/acsomega.4c01387)
Supplement: Supplementary file 1 — ao4c01387_si_001.pdf [file ao4c01387_si_001.pdf]

Supporting Information

Enzymatically Triggered Peptide-Lipid Conjugation of Designed Membrane Active Peptides for Controlled Liposomal Release

*Alexandra Iversen, Johanna Utterström, Robert Selegård, Daniel Aili**

Laboratory of Molecular Materials, Division of Biophysics and Bioengineering, Linköping University, 581 83 Linköping, Sweden.

Corresponding author: daniel.aili@liu.se

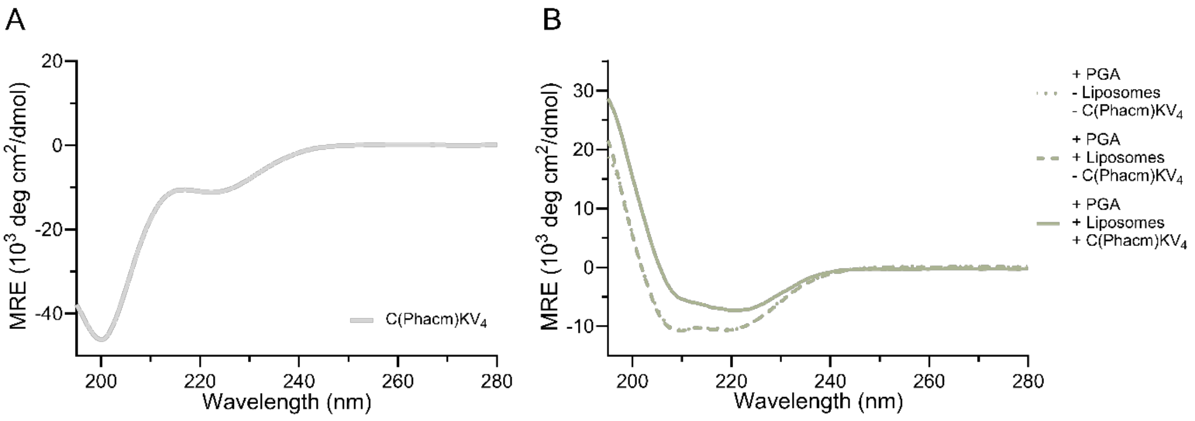

Figure S1. A) CD spectra of 30 µM C(Phacm)KV$_4$ in 0.01 M PB. B) CD spectra of 1.5 µM PGA without (dotted) and with 1.2 mM of 95:5 POPC/MPB vesicles (dashed) and 0.15 µM PGA with 30 µM C(Phacm)KV$_4$ (solid) and 1.2 mM of 95:5 POPC/MPB vesicles after 17.5 hours. All measurements were carried out in 0.01 M PB.

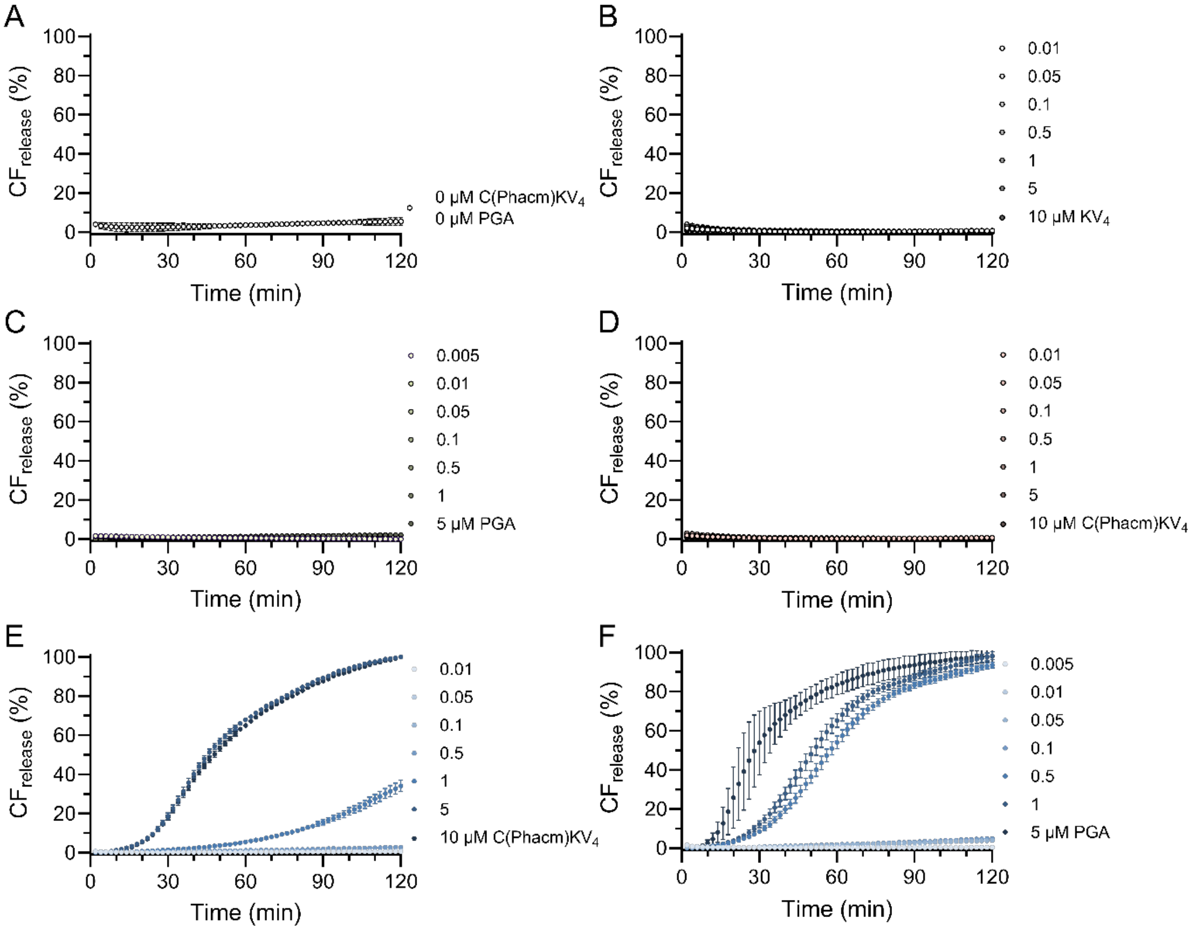

Figure S2. A) CF release kinetics from 40 µM 95:5 POPC:MPB vesicles over 2 hours. B) CF release kinetics from 40 µM 95:5 POPC:MPB vesicles over 2 hours incubation with 0.01 – 10 µM KV_4. C) CF release kinetics from 40 µM 95:5 POPC:MPB vesicles over 2 hours incubation with 0.005 – 5 µM PGA. D) CF release kinetics from 40 µM 95:5 POPC:MPB vesicles over 2 hours incubation with 0.01 – 10 µM C(Phacm)KV_4. E) CF release kinetics from 40 µM 95:5 POPC:MPB vesicles over 2 hours incubation with varying concentrations of C(Phacm)KV_4 (0.01 – 10 µM) and 0.5 µM PGA. F) CF release kinetics from 40 µM 95:5 POPC:MPB vesicles during 2 hours incubation with varying concentrations of PGA (0.005 – 5 µM) and 1 µM C(Phacm)KV_4.

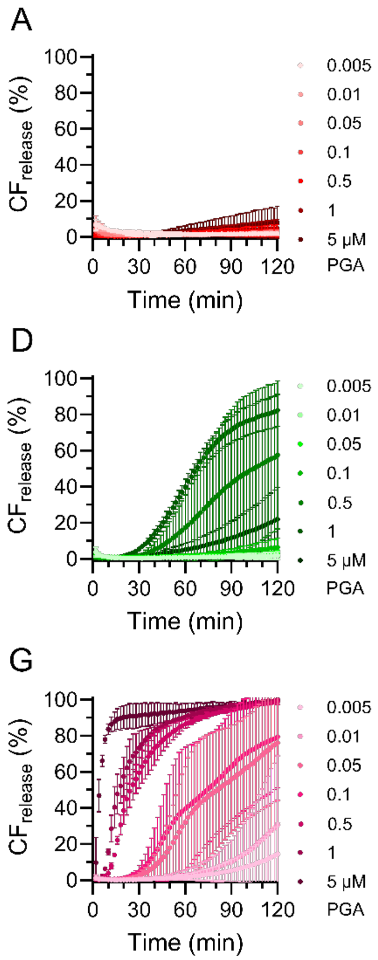

Figure S3. CF release kinetics from 40 μM 95:5 POPC/MPB vesicles over 2 h incubation when incubated with varying concentrations of PGA and (A) 0.01 (B) 0.05 (C) 0.1 (D) 0.5 (E) 1 (F) 5 (G) 10 μM C(Phacm)KV_4.

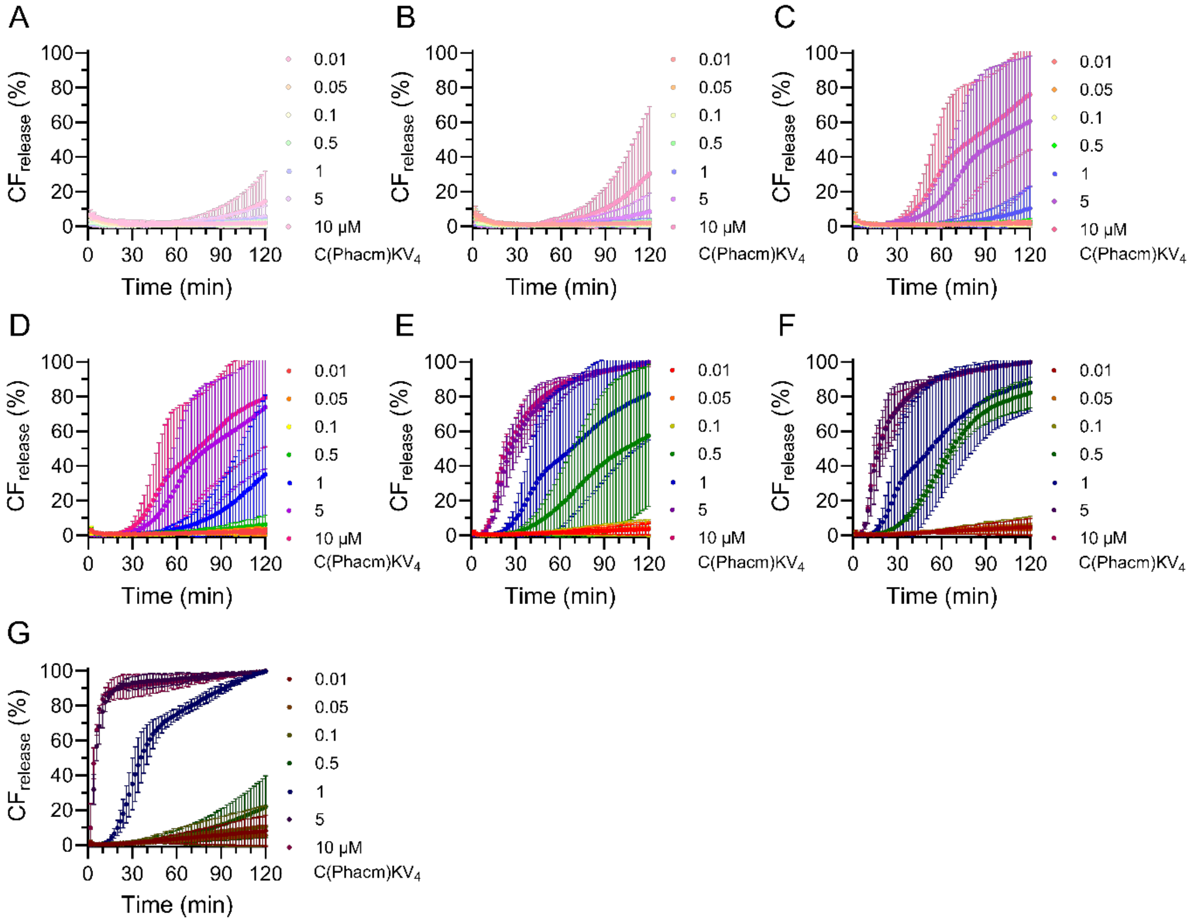

Figure S4. CF release kinetics from 40 µM 95:5 POPC/MPB vesicles over 2 h incubation when incubated with varying concentrations of C(Phacm)KV$_4$ and (A) 0.005 (B) 0.01 (C) 0.05 (D) 0.1 (E) 0.5 (F) 1 (G) 5 µM PGA.

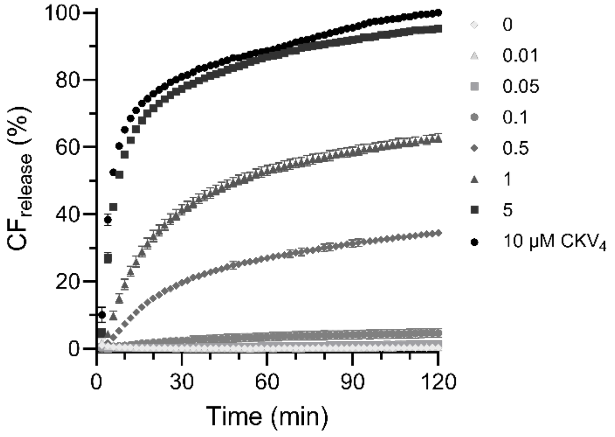

Figure S5. CF release kinetics from 40 μM 95:5 POPC:MPB vesicles over 2 hours incubation with 0 – 10 μM CKV_4.

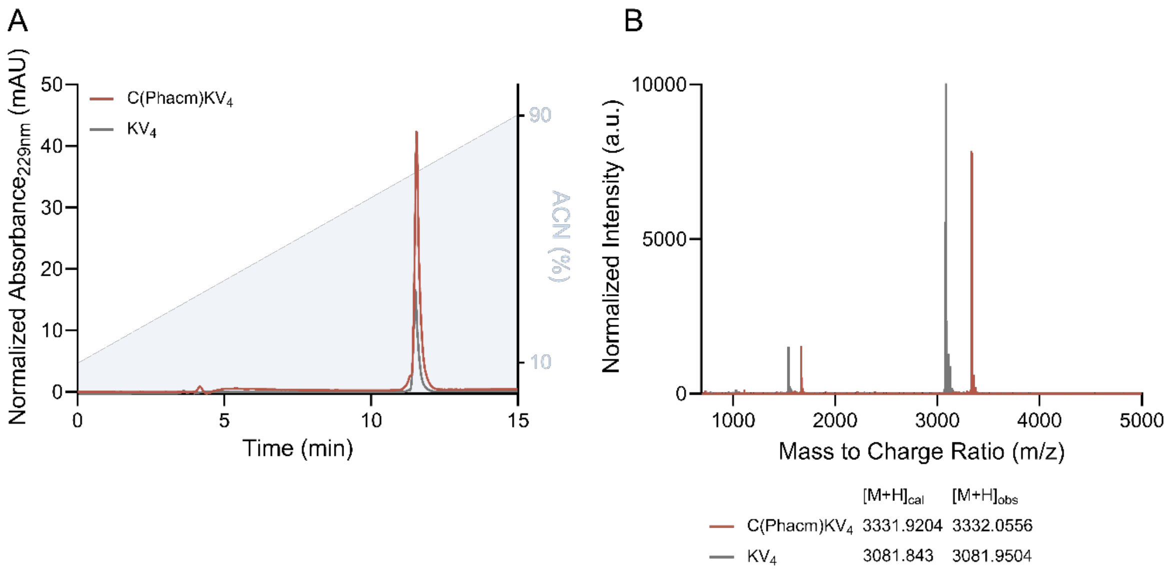

Figure S6. A) Analytical HPLC chromatogram of C(Phacm)KV_4 (pink) and KV_4 (grey) showing peptide purity. Blue graph indicates the aqueous gradient of acetonitrile used. B) Mass spectra of C(Phacm)KV_4 (pink) and KV_4 (grey) acquired by MALDI-TOF MS running in positive reflector mode using α-cyano-4-hydroxycinnamic acid as matrix.
